# Demographic Factors and Aortic Stenosis-Related Death Locations: A Cross-Sectional Analysis

**DOI:** 10.3390/jcm14061969

**Published:** 2025-03-14

**Authors:** Adam Bacon, Hesham Abdalla, Ramzi Ibrahim, Mohamed Allam, Maryam Emami Neyestanak, Ghee Kheng Lim, Xuan Ci Mee, Hoang Nhat Pham, Mahmoud Abdelnabi, Justin Z. Lee, Juan Farina, Chadi Ayoub, Reza Arsanjani, Kwan Lee

**Affiliations:** 1 Department of Medicine, Mayo Clinic, Phoenix, AZ 85054, USA; bacon.adam@mayo.edu (A.B.); abdalla.hesham@mayo.edu (H.A.); 2 Department of Cardiovascular Medicine, Mayo Clinic, Phoenix, AZ 85054, USA; allam.mohamed@mayo.edu (M.A.); lim.gheekheng@mayo.edu (G.K.L.); mee.xuanci@mayo.edu (X.C.M.); abdelnabi.mahmoud@mayo.edu (M.A.); farina.juanmaria@mayo.edu (J.F.); ayoub.chadi@mayo.edu (C.A.); arsanjani.reza@mayo.edu (R.A.); lee.kwan@mayo.edu (K.L.); 3 Department of Epidemiology and Biostatistics, University of Arizona, Tucson, AZ 85721, USA; maryame@arizona.edu; 4 Department of Medicine, University of Arizona, Tucson, AZ 85721, USA; npham917@arizona.edu; 5 Department of Cardiovascular Medicine, Cleveland Clinic, Cleveland, OH 44195, USA; leej67@ccf.org

**Keywords:** aortic stenosis, demographics, social, epidemiology

## Abstract

**Background**: Aortic stenosis (AS) imposes a significant mortality burden. Understanding demographic influences on the location of AS-related death is crucial for advancing equitable end-of-life care. Therefore, we investigated how demographic factors influence the location of death among AS patients in the United States. **Methods**: We completed a cross-sectional study utilizing US mortality data from the CDC’s WONDER database for 2019. All files related to decedents with AS identified as the primary cause of death were obtained, including demographic information and death locations (i.e., inpatient facilities, outpatient/ER facilities, home, or hospice/nursing facilities). Associations between demographic factors (age, sex, race/ethnicity, marital status, and education) and place of death were assessed using multivariable logistic regression models, yielding odds ratios (ORs). **Results**: In 2019, most AS-related deaths occurred in inpatient facilities (38.3%, n = 5062), home (29.2%, n = 3859), or hospice/nursing facilities (28.6%, n = 3775). Higher odds of inpatient death were observed among Black (OR 1.67, *p* < 0.001) and Hispanic individuals (OR 1.91, *p* < 0.001) compared to White decedents. Those aged >85 years were more likely to die at home (OR 1.76, *p* < 0.001) or in hospice/nursing facilities (OR 7.80, *p* < 0.001). Males had increased odds of inpatient death (OR 1.09, *p* = 0.044) but decreased odds of hospice/nursing facility death (OR 0.87, *p* = 0.003). Higher education levels were associated with increased odds of home death (OR 1.33, *p* = 0.023) and decreased odds of hospice/nursing facility death (OR 0.71, *p* = 0.015). **Conclusions**: Demographic factors significantly influence the location of death among AS patients, emphasizing the need for culturally and socioeconomically tailored interventions to promote equitable end-of-life care.

## 1. Introduction

Aortic stenosis (AS) is the second most common valvular disease, affecting approximately 5% of Americans and up to 13.4% of the global population, with prevalence shown to increase exponentially with age [1,2]. When left untreated, AS is associated with varying rates of all-cause mortality: 25% for patients with mild AS, and up to 44% and 42% for those with moderate to severe and severe AS, respectively [3]. Despite therapeutic advancements in aortic valve replacement (AVR) and transcatheter aortic valve replacement (TAVR) [4], AS continues to impose a substantial mortality burden, particularly on elderly patients who often have multiple comorbidities that may preclude them from being procedural candidates [4].

The location of death for patients with AS varies widely and is influenced by factors such as financial means, healthcare access, perceived condition severity, and demographic characteristics. While deaths in inpatient facilities like hospitals and nursing homes have decreased, there has been an increase in deaths occurring at home or in hospice settings [5]. Ethnicity and race further impact a patient’s place of death, with non-White minorities showing a greater tendency to die in the hospital [6]. Socioeconomic strain disproportionately affects these groups, often leading to less confidence in managing conditions at home or attending follow-up with primary care providers [6], thus increasing their likelihood of hospitalization. Sex differences also contribute, with women being less likely to die at home compared to White and male populations [6]. These examples emphasize the significant influence demographic factors have on place of death.

Despite the importance of these influences, limited epidemiological data exist on the location of death among persons with AS. Therefore, we aimed to investigate the impact of demographic characteristics on the locations of AS-related deaths in the United States.

## 2. Methods

Data for this analysis were obtained from the Centers for Disease Control and Prevention (CDC) Wide-ranging Online Data for Epidemiologic Research (WONDER) database, a comprehensive source for county-level mortality and population data across the United States (https://www.cdc.gov/nchs/nvss/mortality_methods.htm, accessed on 23 February 2025). This mortality dataset is derived from death certificates filed across the fifty states and the District of Columbia, excluding nonresidents and fetal deaths. It includes a primary cause of death along with up to twenty secondary causes. Individual states code these data and submit them to the National Center for Health Statistics (NCHS) through the Vital Statistics Cooperative Program or provide death certificates directly to the NCHS for coding.

Using the appropriate International Classification of Diseases, 10th Revision (ICD-10) codes, we collected data on all cases with AS (ICD10: I35.0 and I35.2) listed as the primary cause of death in the year 2019. Place of death was classified in alignment with the WONDER database, categorized as inpatient facilities, outpatient/ER facilities, home, or hospice/nursing facilities. To explore disparities in places of death based on demographic factors, we also queried for additional details such as age group, race/ethnicity, highest level of education, and marital status.

### Statistical Analysis

We analyzed all deaths primarily attributed to AS in 2019, categorizing cases by place of death. For each location, we reported the number of AS-related cases along with percentile ranges within the respective demographic groups. Consequently, this study focused on evaluating the impact of demographic factors on AS-related deaths in 2019 alone. For assessing associations between demographic factors and place of death, multivariable logistic regression analyses were conducted in 2019, which was the latest year available where we may potentially avoid confounding data from the COVID-19 pandemic. The regression models adjusted for factors including age group, sex, ethnicity, race, marital status, and highest level of educational attainment. Standard reference categories included males (compared to females), age groups compared to those under 65 years, Hispanic (compared to non-Hispanic individuals), racial groups (Black, Asian/Pacific Islander, Indian/Alaska Native compared to White), marital statuses (married/widowed/divorced compared to single), and educational attainment (higher education levels compared to 12th grade or less). Each model was adjusted for a specific place of death, generating odds ratios (ORs) and their corresponding 95% confidence intervals (CIs) for comparison. A two-sided *p*-value of <0.05 was considered statistically significant.

Statistical analyses were conducted in RStudio (2023.03.0). Since the data used were publicly accessible and involved deceased individuals, Institutional Review Board approval was not required.

## 3. Results

In 2019, AS was documented as the primary cause of 13,206 deaths. Patient-level data indicate that the majority of these deaths occurred among individuals aged 85 and older (64.4%, 8510 cases), followed by those aged 65–84 (31.3%, 4129 cases). Females accounted for 57.9% (7648 cases) of all deaths, and White individuals comprised a substantial majority at 93.1% (12,301 cases). Most decedents were non-Hispanic (95.6%, 12,621 cases), and approximately half were widowed (53.8%, 7109 cases). The distribution of death locations showed that 5062 deaths (38.3%) occurred in inpatient facilities, 510 (3.9%) in outpatient/ER facilities, 3859 (29.2%) at home, and 3775 (28.6%) in hospice or nursing facilities (Table 1).

### 3.1. Inpatient Mortality

Decedents aged 65–84 had a lower incidence of inpatient death compared to those under 65 (OR 0.82 [0.68–0.99], *p* = 0.04), with an even greater reduction observed among those over 85 (OR 0.31 [0.25–0.37], *p* < 0.001) (Table 2). Male decedents exhibited an increased odds of inpatient death compared to females (OR 1.09 [1.00–1.18], *p* = 0.044). Race also played a significant role as Black (OR 1.67 [1.40–2.00], *p* < 0.001) and Asian/Pacific Islander (OR 1.39 [1.09–1.77], *p* = 0.008) decedents had higher odds of inpatient death compared to White decedents. Hispanic ethnicity was similarly associated with a higher odds of inpatient death (OR 1.91 [1.60–2.28], *p* < 0.001) compared to non-Hispanic individuals. Married decedents were more likely to die in inpatient settings than single individuals (OR 1.25 [1.05–1.49], *p* = 0.013). In contrast, the odds of inpatient death among American Indian/Alaska Native decedents were not significantly different from White decedents, and the highest level of educational attainment did not significantly impact the risk of inpatient death.

### 3.2. Outpatient/Emergency Room Mortality

Age was strongly associated with the risk of death in the outpatient/ER setting, as decedents aged 65–84 were less likely to die in this setting (OR 0.46 [0.34–0.63], *p* < 0.001), with an even further reduction in the odds observed for those over 85 (OR 0.26 [0.19–0.36], *p* < 0.001) compared to decedents under 65 (Table 3). Male decedents showed higher odds of death in outpatient/ER settings compared to females (OR 1.40 [1.15–1.70], *p* = 0.001). Hispanic decedents were also more likely to die in these settings compared to non-Hispanic decedents (OR 2.09 [1.49–2.86], *p* < 0.001), whereas no significant associations were observed for Black or Asian/Pacific Islander populations. However, American Indian/Alaska Native decedents had increased rates of outpatient/ER mortality (OR 2.85 [0.96–6.80], *p* = 0.032) compared to White decedents. Marital status and educational attainment did not significantly affect the likelihood of death in the outpatient/ER setting.

### 3.3. Home Mortality

Age > 85 was associated with a higher likelihood of death at home (OR 1.76 [1.42–2.21], *p* < 0.001) compared to those under 65 (Table 4). Decedents with a doctorate degree were more likely to die at home (OR 1.33 [1.04–1.69], *p* = 0.023), while other educational levels showed no significant effect. Married decedents also had increased odds of home mortality compared to single individuals (OR 1.22 [1.01–1.49], *p* = 0.046). Hispanic decedents had lower odds of dying at home than non-Hispanic decedents (OR 0.72 [0.58–0.88], *p* = 0.002). No significant differences in home mortality were found for Black, American Indian/Alaska Native, or Asian/Pacific Islander decedents compared to White decedents, and sex did not significantly impact the likelihood of dying at home.

### 3.4. Hospice/Nursing Facility Mortality

The likelihood of dying in a hospice or nursing facility increased substantially with age, with an odds ratio of 3.49 [2.45–5.16] (*p* < 0.001) for those aged 65–84 and 7.80 [5.48–11.49] (*p* < 0.001) for those over 85, compared to decedents under 65 (Table 5). Decedents with a doctorate degree were less likely to die in these settings (OR 0.71 [0.53–0.93], *p* = 0.015) compared to those with a high school education or less. Males had a lower odds of death in hospice/nursing facilities than females (OR 0.87 [0.80–0.96], *p* = 0.003). Marital status also influenced the odds, with married (OR 0.54 [0.45–0.66], *p* < 0.001), divorced (OR 0.75 [0.60–0.94], *p* = 0.011), and widowed (OR 0.81 [0.67–0.99], *p* = 0.033) decedents all demonstrating lower likelihoods of dying in hospice/nursing facilities compared to single decedents. Racial and ethnic differences were also evaluated, showing that Black (OR 0.54 [0.43–0.68], *p* < 0.001), American Indian/Alaska Native (OR 0.34 [0.12–0.91], *p* = 0.027), Asian/Pacific Islander (OR 0.53 [0.39–0.72], *p* < 0.001), and Hispanic (OR 0.43 [0.33–0.54], *p* < 0.001) decedents were significantly less likely to die in hospice/nursing facilities compared to White decedents and non-Hispanic decedents, respectively.

## 4. Discussion

Our findings highlight the influence of demographic factors on the place of AS-related deaths in the United States. We identified three key trends: (1) race and ethnicity significantly affect the utilization of hospice or nursing facilities during end-of-life care, (2) sex and marital status are associated with differences in their respective places of AS-related death, (3) individuals with a doctorate degree are more likely to die at home and less likely in hospice or nursing facilities compared to those with minimal educational attainment, and (4) increasing age was significantly associated with a higher odds of hospice/nursing facility or at-home AS-related deaths. Collectively, these findings emphasize the role of individual factors in shaping end-of-life care settings, showing the importance of equitable healthcare access and resources for end-of-life care in the United States.

The 2019 data on AS decedents show significant variation in the place of death, largely influenced by demographic factors. In line with the rising prevalence of AS with age, we found that most AS decedents were aged 85 and older. The likelihood of dying from AS in a hospice or nursing facility also increased significantly with advancing age. While advancements in transcatheter and surgical valve replacement procedures have improved outcomes, many elderly patients are not candidates for these invasive treatments [3]. Younger patients, particularly those under 65, were more likely to die in outpatient or emergency settings, potentially due to the higher prevalence of bicuspid aortic valve in the younger populations along with the acuity of their presentations, which often leads to rapid disease progression and acute presentations [7].

In general, non-white minorities show a preference for inpatient deaths [6]. This pattern held among AS decedents, with Black, Asian/Pacific Islander, and Hispanic individuals demonstrating higher odds of inpatient death compared to White individuals. Racial and ethnic minorities were also shown to have reduced odds of hospice/nursing facility utilization. Factors such as limited education, financial strain, structural racism, and higher religious involvement, which has been shown to increase preferences for inpatient care and life-prolonging measures, may contribute to this trend [8,9,10,11]. Furthermore, misconceptions regarding the utility of hospice care have been previously shown among racial and ethnic minority groups. Socioeconomic disparities are likely to contribute to the disparate end-of-life care utilization, emphasizing the importance of incorporating measures to appropriately educate and assess eligibility of planned end-of-life care measures [12].

Our findings show that males are at greater risk of inpatient death than females, while being less likely to die in hospice or nursing facilities. Males have been previously shown to less likely engage in preventative care and may delay seeking medical attention, potentially leading to more acute presentations requiring inpatient care [13]. In regards to AS, late presentation or diagnosis may lead to worse outcomes including sudden cardiac death. Males have also been shown to participate in end-of-life care planning at lesser rates than their female counterparts, which may contribute to lower hospice utilization [14]. Married decedents had higher rates of inpatient and at-home deaths than single individuals, which may potentially be explained by a multitude of factors [15,16,17,18]. Prior studies have shown potentially better health behaviors among married individuals, which was associated with reduced rates of cardiovascular comorbidities [15,16,17,18]. A spouse can play a crucial role in supporting the transition from inpatient or nursing facility care to home, providing consistent assistance during this period. Additionally, larger household sizes and the presence of multiple family members can further enhance the support available for at-home care services [18].

Educational attainment did not significantly influence the risk of death in inpatient or outpatient/ER settings. However, those with a doctorate degree had higher odds of dying at home and lower odds of death in nursing or hospice facilities. This aligns with research on patients with cancer, suggesting that individuals with higher education may prefer home death [19], potentially due to increased financial resources helping facilitate home-based end of life care without the need for hospice or inpatient facilities. However, we are unable to differentiate if these “home” deaths were incorrectly classified as a “home” death during utilization of hospice services. Nonetheless, prior studies have also shown that higher educated groups are often against aggressive end-of-life treatment, potentially due to their emphasis on autonomy and self-determination over their final stages of life [20].

Within the era of advancements using transcatheter aortic valve replacement, candidacy for aortic valvular replacements has significantly increased. However, AS remains a major cause of mortality, especially in those that are not procedural candidates, late diagnosis, or have limited access to care. For example, symptomatic patients with severe AS have a high mortality risk, facing a 40–50% mortality rate at one year and up to 80% by three years [21,22]. This sharp increase emphasizes the need for early symptom management and clear goal-setting in care. Exercise echocardiography may help with prognostication in these patients. One prospective, observational study that enrolled 148 patients with isolated asymptomatic aortic stenosis found that 36 of these patients had an abnormal exercise stress test and were ultimately referred to surgery [23]. Among the 112 patients with normal findings on the exercise test, 38 patients had abnormal exercise echocardiography scores (mean pressure gradient >20 mmHg, peak systolic pulmonary artery pressure at peak exercise >60 mmHg). However, abnormal echocardiography parameters based on the mean pressure gradient or peak systolic pulmonary artery pressure did not predict occurrence of aortic stenosis-related events. However, another prospective, observational study evaluated the role of exercise stress echocardiography in 90 patients with mild and moderate aortic stenosis [24]. In this study, patients who developed symptoms during exercise, an increase of at least 15 mmHg in mean transaortic pressure gradient, significant electrocardiogram changes, or an exercise-induced pseudo-normalization of the E/A ratio had a higher rate of cardiac events within one year.

Palliative care, which focuses on improving quality of life for patients with serious illnesses, is often misunderstood as solely “end-of-life care.” This misconception can lead to distrust in palliative care providers and the intentions of those recommending it. While palliative care has expanded across the United States, its adoption has been slower among non-White minority populations [25]. Misunderstandings around palliative care, such as viewing it as the cessation of life-prolonging treatments or solely “comfort care”, akin to hospice, may shape these groups’ preferences for place of death and prevent them from fully benefiting from palliative services [26]. Addressing these misconceptions can help patients better understand palliative care, allowing them to make informed choices and fostering a sense of autonomy in end-of-life decisions related to AS.

Our data emphasize the complexities of end-of-life care in AS patients and reveal significant disparities based on demographic factors. These findings highlight the need to prioritize equitable and accessible end-of-life care for all individuals. Addressing these challenges requires a strong emphasis on educating minority groups, who are less likely to use these services, about advanced care planning and end-of-life options. Culturally tailored interventions using bilingual, easy-to-understand materials have shown promise in raising awareness of end-of-life decision-making within ethnic minority communities [27,28]. Community-based initiatives are also essential to ensure that palliative and hospice services reach underrepresented populations. Tools like geographic mapping can support the strategic distribution of resources, helping to establish specialized care centers in areas where they will have the most impact [7]. By leveraging demographic data on AS mortality with geospatial mapping, policymakers can make more informed decisions on resource allocation, thus incentivizing efforts to address these disparities. Finally, further investigation into the mechanisms driving these demographic differences is crucial, as current data remain limited. A deeper understanding will enable more targeted interventions, ultimately helping to reduce these disparities and improve end-of-life care for AS patients across diverse communities.

This analysis has inherent limitations related to the CDC’s death certificate data, which restrict our ability to determine if the reported place of death aligned with the patient’s wishes. Additionally, the death certificate identifies aortic stenosis as the primary cause of death but does not capture other medical comorbidities that might have prevented patients from receiving life-prolonging interventions, thus affecting survival outcomes. Another limitation is the CDC’s classification of hospice as a place of death, which may not account for all patients who passed away under hospice care in settings such as inpatient facilities or at home. While we considered several demographic factors, some of these data, particularly ethnicity, are subject to change. For instance, census data show that 34% of respondents reported a different ethnicity within a two-year period [29]. Given the fluid nature of such data, conclusions drawn from these variables may contain inherent inaccuracies. Lastly, we are unable to assess whether the decedent’s respective location of death aligned with their end-of-life care preference.

## 5. Conclusions

This study highlights significant disparities in the locations of death among AS patients, influenced by demographic and socioeconomic factors. These findings emphasize the importance of understanding the underlying mechanisms driving these inequities to better support patients in achieving end-of-life experiences aligned with their personal preferences and beliefs.

## Figures and Tables

**Table 1:** Aortic stenosis decedent characteristics in 2019. This table depicts the patient-level demographic data of all decedents related to aortic stenosis as the primary problem in their death certificate in 2019, stratified by place of death.

Population	Variable	Overall	Inpatient	Outpatient	Home	Hospice/Nursing Facility
		13,206	5062	510	3859	3775
Age groups (%)	≤64 years	567 (4.3)	351 (6.9)	69 (13.5)	115 (3.0)	32 (0.8)
	65–84 years	4129 (31.3)	2263 (44.7)	218 (42.7)	916 (23.7)	732 (19.4)
	≥85 years	8510 (64.4)	2448 (48.4)	223 (43.7)	2828 (73.3)	3011 (79.8)
Sex (%)	Female	7648 (57.9)	2699 (53.3)	225 (44.1)	2274 (58.9)	2450 (64.9)
	Male	5558 (42.1)	2363 (46.7)	285 (55.9)	1585 (41.1)	1325 (35.1)
Race (%)	White	12,301 (93.1)	4605 (91.0)	461 (90.4)	3615 (93.7)	3620 (95.9)
	Black	566 (4.3)	303 (6.0)	28 (5.5)	139 (3.6)	96 (2.5)
	American Indian	44 (0.3)	22 (0.4)	5 (1.0)	12 (0.3)	5 (0.1)
	Asian/Pacific Islander	295 (2.2)	132 (2.6)	16 (3.1)	93 (2.4)	54 (1.4)
Marital Status (%)	Single	672 (5.1)	295 (5.8)	35 (6.9)	160 (4.1)	182 (4.8)
	Divorced	1350 (10.2)	638 (12.6)	68 (13.3)	318 (8.2)	326 (8.6)
	Married	4075 (30.9)	1891 (37.4)	220 (43.1)	1159 (30.0)	805 (21.3)
	Widowed	7109 (53.8)	2238 (44.2)	187 (36.7)	2222 (57.6)	2462 (65.2)
Education (%)	12th grade or less	2264 (17.1)	874 (17.3)	90 (17.6)	644 (16.7)	656 (17.4)
	High school/GED/some college	7511 (56.9)	2899 (57.3)	276 (54.1)	2134 (55.3)	2202 (58.3)
	Associates degree	774 (5.9)	300 (5.9)	28 (5.5)	233 (6.0)	213 (5.6)
	Bachelor degree	1587 (12.0)	584 (11.5)	74 (14.5)	500 (13.0)	429 (11.4)
	Master degree	719 (5.4)	266 (5.3)	31 (6.1)	224 (5.8)	198 (5.2)
	Doctorate degree	351 (2.7)	139 (2.7)	11 (2.2)	124 (3.2)	77 (2.0)
Hispanic (%)	Non-Hispanic	12,621 (95.6)	4735 (93.5)	460 (90.2)	3732 (96.7)	3694 (97.9)
	Hispanic	585 (4.4)	327 (6.5)	50 (9.8)	127 (3.3)	81 (2.1)

**Table 2:** Inpatient aortic stenosis-related death, 2019. Multivariable logistic regression model evaluating the impact of demographic factors on their association with inpatient aortic stenosis related deaths.

Independent Variables	Odds Ratios	95% CI	*p*-Value
Intercept	1.14	0.90–1.45	0.266
Age group–65–84 y/o	0.82	0.68–0.99	0.043
Age group—≥85 y/o	0.31	0.25–0.37	<0.001
Education—high school/GED/some college	1.04	0.94–1.15	0.471
Education—associates	1.01	0.85–1.21	0.872
Education—bachelors	0.94	0.82–1.08	0.373
Education—masters	0.96	0.80–1.16	0.691
Education—doctorate	1.04	0.82–1.33	0.727
Sex—male	1.09	1.00–1.18	0.044
Marital status—divorced	1.20	0.99–1.46	0.068
Marital status—married	1.25	1.05–1.49	0.013
Marital status—widowed	0.97	0.82–1.16	0.772
Hispanic	1.91	1.60–2.28	<0.001
Race—Black	1.67	1.40–2.00	<0.001
Race—Indian/Alaska Native	1.50	0.80–2.78	0.200
Race—Asian/Pacific Islander	1.39	1.09–1.77	0.008
Observations = 13,206			
Coefficient of discrimination = 0.081			

Standard reference categories included males (compared to females), age groups compared to those under 65 years, Hispanic (compared to non-Hispanic individuals), racial groups (Black, Asian/Pacific Islander, Indian/Alaska Native compared to White), marital statuses (married/widowed/divorced compared to single), and educational attainment (higher education levels compared to 12th grade or less). Abbreviations: CI = confidence interval, y/o = years old.

**Table 3:** Outpatient/emergency room aortic stenosis-related death, 2019. Multivariable logistic regression model evaluating the impact of demographic factors on their association with outpatient/emergency room aortic stenosis death.

Independent Variables	Odds Ratios	95% CI	*p*-Value
Intercept	0.08	0.05–0.12	<0.001
Age group—65–84 y/o	0.46	0.34–0.63	<0.001
Age group—≥85 y/o	0.26	0.19–0.36	<0.001
Education—high school/GED/some college	1.01	0.79–1.31	0.939
Education—associates	0.97	0.61–1.48	0.879
Education—bachelors	1.24	0.90–1.72	0.187
Education—masters	1.18	0.76–1.79	0.450
Education—doctorate	0.77	0.38–1.41	0.432
Sex—male	1.40	1.15–1.70	0.001
Marital status—divorced	1.19	0.78–1.84	0.439
Marital status—married	1.28	0.89–1.91	0.203
Marital status—widowed	0.96	0.65–1.46	0.841
Hispanic	2.09	1.49–2.86	<0.001
Race—Black	1.19	0.78–1.74	0.402
Race—Indian/Alaska Native	2.85	0.96–6.80	0.032
Race—Asian/Pacific Islander	1.46	0.83–2.37	0.156
Observations = 13,206			
Coefficient of discrimination = 0.018			

Standard reference categories included males (compared to females), age groups compared to those under 65 years, Hispanic (compared to non-Hispanic individuals), racial groups (Black, Asian/Pacific Islander, Indian/Alaska Native compared to White), marital statuses (married/widowed/divorced compared to single), and educational attainment (higher education levels compared to 12th grade or less). Abbreviations: CI = confidence interval, y/o = years old.

**Table 4:** Home aortic stenosis-related death, 2019. Multivariable logistic regression model evaluating the impact of demographic factors on their association with home aortic stenosis death.

Independent Variables	Odds Ratios	95% CI	*p*-Value
Intercept	0.24	0.18–0.32	<0.001
Age group—65–84 y/o	1.03	0.83–1.29	0.783
Age group—≥85 y/o	1.76	1.42–2.21	<0.001
Education—high school/GED/some college	0.98	0.88–1.09	0.722
Education—associates	1.08	0.90–1.29	0.410
Education—bachelors	1.13	0.98–1.31	0.089
Education—masters	1.12	0.93–1.34	0.250
Education—doctorate	1.33	1.04–1.69	0.023
Sex—male	0.98	0.90–1.07	0.634
Marital status—divorced	0.99	0.80–1.24	0.951
Marital status—married	1.22	1.01–1.49	0.046
Marital status—widowed	1.17	0.97–1.42	0.112
Hispanic	0.72	0.58–0.88	0.002
Race—Black	0.88	0.72–1.07	0.221
Race—Indian/Alaska Native	0.98	0.48–1.87	0.957
Race—Asian/Pacific Islander	1.09	0.84–1.39	0.510
Observations = 13,206			
Coefficient of discrimination = 0.017			

Standard reference categories included males (compared to females), age groups compared to those under 65 years, Hispanic (compared to non-Hispanic individuals), racial groups (Black, Asian/Pacific Islander, Indian/Alaska Native compared to White), marital statuses (married/widowed/divorced compared to single), and educational attainment (higher education levels compared to 12th grade or less). Abbreviations: CI = confidence interval, y/o = years old.

**Table 5:** Hospice/nursing facility aortic stenosis-related death, 2019. Multivariable logistic regression model evaluating the impact of demographic factors on their association with hospice/nursing facility aortic stenosis death.

Independent Variables	Odds Ratios	95% CI	*p*-Value
Intercept	0.11	0.07–0.16	<0.001
Age group—65–84 y/o	3.49	2.45–5.16	<0.001
Age group—≥85 y/o	7.80	5.48–11.49	<0.001
Education—high school/GED/some college	0.97	0.87–1.08	0.590
Education—associates	0.90	0.74–1.09	0.275
Education—bachelors	0.90	0.77–1.04	0.146
Education—masters	0.89	0.73–1.07	0.221
Education—doctorate	0.71	0.53–0.93	0.015
Sex—male	0.87	0.80–0.96	0.003
Marital status—divorced	0.75	0.60–0.94	0.011
Marital status—married	0.54	0.45–0.66	<0.001
Marital status—widowed	0.81	0.67–0.99	0.033
Hispanic	0.43	0.33–0.54	<0.001
Race—Black	0.54	0.43–0.68	<0.001
Race—Indian/Alaska Native	0.34	0.12–0.81	0.027
Race—Asian/Pacific Islander	0.53	0.39–0.72	<0.001
Observations = 13,206			
Coefficient of discrimination = 0.060			

Standard reference categories included males (compared to females), age groups compared to those under 65 years, Hispanic (compared to non-Hispanic individuals), racial groups (Black, Asian/Pacific Islander, Indian/Alaska Native compared to White), marital statuses (married/widowed/divorced compared to single), and educational attainment (higher education levels compared to 12th grade or less). Abbreviations: CI = confidence interval, y/o = years old.

## Data Availability

The data presented in this study are openly available in CDC WONDER, at link: https://wonder.cdc.gov/mcd.html, accessed on 23 February 2025.
